# Live Imaging of Primary Neurons in Long-Term Cryopreserved Human Nerve Tissue

**DOI:** 10.1523/ENEURO.0388-21.2021

**Published:** 2021-11-30

**Authors:** Marina Fortea, Piyush Jain, Ingrid Demedts, Jan Tack, Tim Vanuytsel, Carla Cirillo, Pieter Vanden Berghe

**Affiliations:** 1 Laboratory for Enteric Neuroscience (LENS), Translational Research Center for Gastrointestinal Disorders, Department of Chronic Diseases and Metabolism, Katholieke Universiteit Leuven, Leuven 3000, Belgium; 2 Translational Research Center for Gastrointestinal Disorders, Department of Chronic Diseases and Metabolism, Katholieke Universiteit Leuven, Leuven 3000, Belgium; 3 Department of Gastroenterology and Hepatology, University Hospitals Leuven, Leuven 3000, Belgium; 4 Toulouse NeuroImaging Center (ToNIC), Inserm, University Paul Sabatier, Toulouse 31024, France; 5 Cell and Tissue Imaging Cluster, Katholieke Universiteit Leuven Core Facility, Leuven 3000, Belgium

**Keywords:** cryopreservation, enteric nervous system, human primary neuron, life imaging

## Abstract

Tissue cryopreservation provides a convenient solution for tackling one of the major problems in neuroscience research, namely, the scarce availability of human nerve tissues, especially if needed alive. While brain tissue can be used only postmortem, live nerve tissue can reasonably well be harvested from the periphery. A valuable source of primary neurons is the intestine, which compared with brain has the advantage to be safely accessible via endoscopy. The nerve tissue innervating the intestine (the enteric nervous system; ENS) can be sampled with regular endoscopic biopsy forceps and remains viable for multiple physiological and immunohistochemical tests, as previously demonstrated. Here, we present a method to preserve, over longer periods of time, human primary neurons contained in these biopsies. The use of a cryoprotective agent and the application of controlled cooling revealed to be crucial to properly store the nerve tissue and to enable functional measurements after thawing. These primary neurons were evaluated for functionality (live imaging) and morphology (histology) up to one year after cryopreservation. Calcium (Ca^2+^) imaging indicated that human primary neurons remained viable and responded to selective stimulations (serotonergic and nicotinic agonists) after cryopreservation. Additionally, immunohistochemistry performed with specific neuronal markers showed that nerve structure and neuronal morphology were retained, with no signs of cellular damage. In this study, we demonstrate that the human ENS is a realistic source of primary neurons, which can be successfully preserved over long times and as such can be exploited both for gastrointestinal-specific as well as for general neuroscience research.

## Significance Statement

We describe a novel protocol for live imaging of human primary neurons after long-term cryopreservation. This provides a meaningful advance in neuroscience, as it enables sharing live tissues between research centers for a variety of experiments. As such, this technique will broaden research networks and facilitate collaborations between institutes worldwide contributing with different expertise.

## Introduction

A key requirement in biomedical, and particularly in translational research, is to adequately test findings derived from cell or animal models in a human setting. Therefore, meeting the demand for live human tissues is the prime challenge for truly advancing translational studies. Given their limited availability, means of preserving viable human tissues such as cryopreservation will greatly facilitate their accessibility and broadly expand the possibilities for their experimental use. From a practical point of view, working with human samples is not always straightforward. It usually requires an intricate path, starting from obtaining samples, which involves ethical concerns, specific hospital facilities, and invasive procedures, followed by the complexity of preserving the samples in correct conditions for the planned experimental testing. Moreover, working with human tissue is inherently complex because of biological variability, generated by, among others, age, sex, disease, environment or stress. Despite all the advances in the biomedical field, translational research is still a complex process, and substantial effort is needed to successfully use human tissue in research. In this context, the establishment of tissue biobanking is priceless for clinical and research applications, especially as it allows performing specific assays in research centers which are not directly associated with a hospital facility.

Since the last century, cryopreservation has been widely used to preserve cells and tissues and, by reducing their decay under extremely cold conditions, keep them alive while maintaining their natural properties. With the discovery of cryoprotective agents, this technique has successfully been used to preserve single cells, but its success rate tends to decrease if used for more complex tissues (Karlsson and Toner, 1996; Bojic et al., 2021). Nowadays, routine procedures efficiently applied to blood, bone marrow cells and embryos do exist (Day and Stacey, 2008; Bakhach, 2009). However, the cryopreservation of human neuronal tissue faces more difficulties. So far, cryopreservation strategies have been applied to neuronal cells and tissue, isolated either from adult or embryonic animals (Uemura and Ishiguro, 2015; Liu and Pan, 2016; Talevi et al., 2016), yet with low or modest success. The tissue used in these studies was the sciatic nerve, isolated from the peripheral nervous system, which served as a source of stem cells used for transplantation or regeneration purposes (Day et al., 2017; Huang et al., 2018; González Porto et al., 2019). In the central nervous system (CNS), two pioneering studies were performed to achieve short-term or long-term cryopreservation of rodent primary neurons (Kawamoto and Barrett, 1986) and human and mouse oligodendrocytes (Seilhean et al., 1996), and to identify possible harmful factors affecting the cryopreservation process (Kawamoto and Barrett, 1986). Notably, tissue from the human CNS is arduous to collect and for reasons of safety and impact not ethical. Thus, successful cryopreservation procedures of human nerve tissue remain highly challenging. Besides the CNS, the enteric nervous system (ENS) is a valuable source of nerve tissue. Different from the brain, the ENS is easily accessible, via routine endoscopy with very low risk for patients. Small intestinal biopsies (∼5–7 mm^2^) contain submucous plexus, a nerve layer that contains a sufficient number of small ganglia comprising neurons and glial cells within them. The possibility to use the human ENS, taken from living subjects, for structural and immunohistochemical analysis has been shown previously (Lebouvier et al., 2008; Pouclet et al., 2012). Moreover, also functional assays were proven possible. A series of studies have now demonstrated the value of the submucosal plexus (SMP) obtained from intestinal biopsies, which, in combination with live microscopic imaging, can be used to assess human neuron and glial cell function (Cirillo et al., 2013, 2015; Wouters et al., 2016; Desmet et al., 2017). Human neurons were functionally and morphologically characterized in healthy and disease (Cirillo et al., 2013) conditions, and this live imaging approach was also employed to evaluate peripheral neurodegeneration in patients with Parkinson’s disease (Desmet et al., 2017). However, the experiments in these studies had to be strictly timed, since the tissue needed to be processed within the same day of collection. Hence, the obvious next step to improve and expand the use of human ENS tissue is to make preservation of these biopsies feasible.

Here, we present a successful protocol to cryopreserve for prolonged time (up to one year), nerve tissues isolated from the human ENS, which, after thawing can still be used for live microscopic imaging.

## Materials and Methods

### Subjects and endoscopy

**Figure 1.**
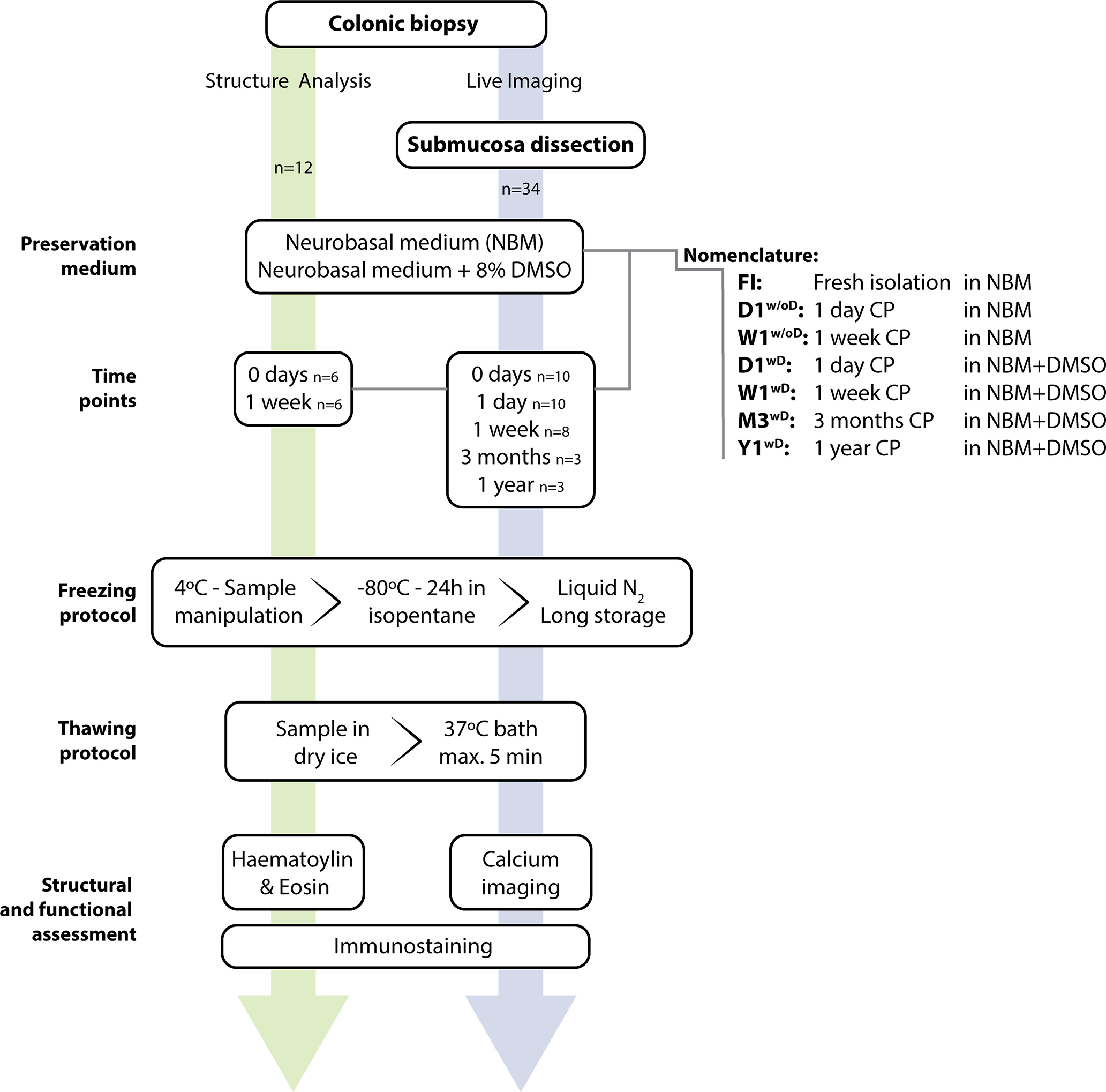
Experimental design and work-flow. Submucous plexus viability was analyzed in six groups of tissues, which were stored in different cryopreserving conditions. Structural and neuron numbers were assessed through H&E and immunostainings, and neuronal viability with Ca^2+^ imaging. The chart also indicates the number of biopsies (n) subjected to each experimental procedure. CP, cryopreservation; FI, fresh isolation; DMSO, Dimethyl sulfoxide; D, day; W, week; M, month; Y, year; NBM, Neurobasal medium.

Colonic mucosal biopsies (Fig.1) were obtained from 34 patients (15 men, mean age 60.9 ± 9.8 and 19 women, mean age 59.36 ± 14.09), who were referred to the endoscopy unit of University Hospitals Leuven for screening colonoscopy. The subjects included in the study were undergoing endoscopy for cancer screening. After obtaining signed informed consent from each subject, four colonic biopsies were taken using standard biopsy forceps (Micro-Tech, Nanjing, NBF01-11123180) by experienced endoscopists (J.T., I.D., and T.V.). The study protocol (S57517-ML11373, 2015) was approved by the Ethics Committee of University Hospitals Leuven. No colonic tumors, colonic polyps >1 cm, or colitis were observed during the course of the endoscopy.

### Sample processing

**Figure 2.**
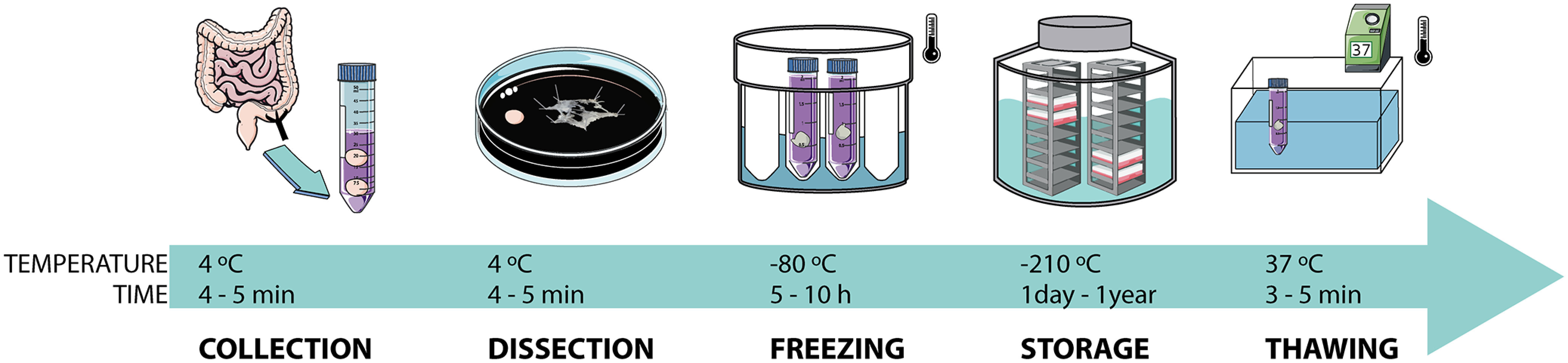
Scheme of protocol for sample processing. Biopsies were collected and placed at 4°C for no more than 30 min before starting the dissection. Once the submucous plexus is dissected, it is placed in NBM with 8% DMSO in isopentane and transferred to a −80°C freezer for 5–10 h. Biopsies are then, for longer storage, placed in liquid nitrogen for up to one year. Thawing was quickly performed, once ice is melted biopsy is changed into fresh NBM at 37°C. NBM, Neurobasal medium.

Colonic mucosal biopsies from left colon were collected and transferred in a tube filled with 5 ml of ice-cold NBM medium [1% penicillin/streptomycin (Sigma, 100×, P4333), 1% GlutaMAX (Invitrogen, 100×, 35050061)], which was maintained at 4°C in a Nalgene Labtop cooler (Nalgene, 5116-1600; Fig. 2).

Once collected, biopsies were directly cryopreserved or dissected within 1 h to obtain the SMP (Cirillo et al., 2013). Briefly, the biopsies were transferred in a Sylgard-lined Petri dish and then carefully pinned flat and dissected with watchmaker’s forceps under a stereomicroscope. Biopsies were continuously perfused with oxygenated (95% oxygen/5% carbon dioxide) ice-cold Krebs solution.

### Cryopreservation optimization strategy

Freezing and thawing protocols of dissected biopsies were amended, aiming for optimal viability after thawing. For this optimization we used: different media complementation [Neurobasal medium (NBM), with or without dimethyl sulfoxide (DMSO)], different storage/freezing conditions (4°C, –20°C, –80°C, and liquid nitrogen), different freezing speeds (in the presence or absence of isopentane), and different periods (from 1 day up to one year) of storage

Temperature changes were monitored using a K-type Peltier element that was immersed into the cryotube down to the bottom where the biopsy would be. A digital multimeter (UNI-T, UT71D, Reichelt Electronics) was used to read-out and digitize the temperature profiles. The tubes were transferred to a −80°C freezer for all conditions analyzed: (1) water, (2) NBM, (3) NBM with DMSO either with or without controlling the cooling speed using a StrataCooler (isopentane container). The digital multimeter recorded the change in temperature over time (every 30 s) during the freezing phase of cryopreservation process (Fig. 2). The temperature was recorded for 110 min or until −60°C were reached. To monitor the thawing process, the cryotubes with the thermo sensor frozen inside were transferred to a water bath (Memmert) maintained at 37°C, during which the temperature was recorded until 37°C was reached. For both freezing and thawing procedures, each measurement was repeated three times and values were averaged.

### Tissue cryopreservation

The schematic design of the study is shown in Figure 1. Briefly, colonic biopsies were either directly preserved (cryopreserved or paraffin embedded) or dissected before cryopreservation, to obtain the nerve tissue, consisting of connective tissue with the SMP embedded within (Cirillo et al., 2013). These tissues were immersed in NBM (with or without DMSO, depending on the condition) and transferred to a −80°C freezer using a StrataCooler. Afterwards, the cryotubes were transferred to a liquid nitrogen (N_2_) tank (Taylor Whartron, LS3000) for long-term storage. Thawing was done as described above, using a water bath at 37°C.

Based on the technical measurements, the following biologically relevant conditions, specified in Table 1, were defined aiming to compare tissue viability.

**Table 1:** Biological conditions assessed for tissue viability

Abbreviation	Cryopreservation	NBM complemented with DMSO
FI	Freshly isolated tissue	No
D1*^w^*^/^*^o^*^D^	1 day cryopreserved	No
W1*^w^*^/^*^o^*^D^	1 week cryopreserved	No
D1*^w^*^D^	1 day cryopreserved	With 8% DMSO
W1*^w^*^D^	1 week cryopreserved	With 8% DMSO
M3*^w^*^D^	3 months cryopreserved	With 8% DMSO
Y1*^w^*^D^	1 year cryopreserved	With 8% DMSO

NBM, Neurobasal medium; DMSO, Dimethyl sulfoxide

Tissue structure and cellular morphology was analyzed with hematoxylin and eosin (H&E) and immunostainings; neuronal viability was evaluated by calcium (Ca^2+^) imaging.

### H&E and immunostaining

For morphologic evaluation of whole-mount biopsies after cryopreservation, tissue sections (5 μm) were processed following the standard procedures of the Pathology department of Universitair Ziekenhuis (UZ) Leuven. For H&E staining, sections were processed by exposing the sample to xylene, 100% ethanol, hematoxylin, alcohol, lithium carbonate, eosin, 70% ethanol, 100% ethanol and xylene, in a series. H&E-stained sections were microscopically (Olympus BX41) visualized and examined for morphologic integrity by an experienced researcher. At least three sections from two biopsies per subject were blindly evaluated in random fashion and scores were assigned using a qualitative scale designed by McGowan and colleagues to grade histologic features of colorectal biopsies (McGowan et al., 2012). Briefly, the histopathology grading scale: (1) Intact epithelium, maintained lamina propria, mucin visible within gland, nuclei stained blue; (2) detached epithelium, maintained lamina propria, mucin visible within glands, nuclei stained blue; (3) detached epithelium, disaggregated lamina propria, mucin visible within glands, nuclei stained blue; (4) mostly detached/lost epithelium, disaggregated lamina propria, no mucin visible within glands, karyorrhectic nuclei >30%; (5) mostly detached/lost epithelium, disaggregated lamina propria, no mucin visible within glands, karyorrhectic nuclei >75%; and (6) lost epithelium, minimal cellular remnants, mostly structural elements remaining.

For immunohistochemical staining, sections were pretreated for 20 min with epitope retrieval solution (Tris/EDTA at pH 9; Leica) and then incubated for 5 min in peroxidase block (part of BOND Polymer refine detection kit, Leica). Sections were incubated for 30 min with primary FLEX Monoclonal Mouse Anti-Human Neuron-Specific Enolase, Clone BBS/NC/VI-H14 (M0873, Dako), ready-to-use (RTU), followed by rabbit anti-mouse postprimary linker (part of BOND Polymer refine detection kit, Leica) for 20 min, HRP-labeled goat anti-rabbit secondary antibody (part of BOND Polymer refine detection kit, Leica), RTU, and then incubated with DAB (part of BOND Polymer refine detection kit, Leica) for 10 min. Sections were then incubated for 30 min with primary FLEX polyclonal rabbit anti-S100 (GA504, Dako), RTU, followed by rabbit anti-mouse postprimary linker (part of BOND Polymer refine detection kit, Leica), for 20 min, AP-labeled goat anti-rabbit secondary antibody (part of BOND Polymer refine detection kit, Leica), RTU, for 20 min and then incubated with FAST red (part of BOND Polymer refine detection kit, Leica) for 10 min. Representative images were acquired with for bright field under epifluorescence microscope, processed (ImageJ 2.0.0-rc-54/1.52d) and adjusted for brightness and contrast.

### Live Ca^2+^ imaging of nerve tissue

After thawing, the cryopreservation medium was removed in a single-step and tissues were put in freshly prepared Krebs solution (120.9 mm NaCl, 5.9 mm KCl, 1.2 mm MgCl_2_, 2.5 mm CaCl_2_, 11.5 mm glucose, 14.4 mm NaHCO_3_, and 1.2 mm NaH_2_PO_4_) previously oxygenated (95% oxygen/5% carbon dioxide). In the case of whole mount specimen, after thawing the biopsy was dissected to obtain the SMP, as described above.

Live imaging was performed at day 0 (FI SMP) or at the different time points after cryopreservation, as indicated in Figure 1. The nerve tissue was transferred to a recording chamber for Ca^2+^ imaging (volume of the chamber: 3 ml) and perfused with room temperature (RT) solution. The nerve tissue was gently stretched and pinned flat and incubated with Krebs solution containing the fluorescent Ca^2+^ indicator Fluo-4 AM (1 μm, Invitrogen) mixed with 0.01% Cremophor EL surfactant agent (Fluka Chemika) for 20 min at RT and under gentle orbital shaking. The tissue was then washed three times for 10 min with Krebs solution. The chamber was transferred to an upright Zeiss Examiner microscope equipped with a 20× (NA 1) water dipping lens and coupled to a monochromator (Poly V) and cooled CCD camera (Imago QE), both from TILL Photonics.

The isolated SMP were kept continuously oxygenated (95% oxygen/5% carbon dioxide). After Fluo-4 loading, the chamber was placed on the stage of an upright Zeiss Examiner microscope. Changes in intracellular Ca^2+^ concentration were measured in response to high-K^+^ (75 mm, 10 s), serotonin (5-HT; 10 μm, 20 s, Sigma), or dimethylphenylpiperazinium (DMPP; 10 μm, 20 s, Fluka Chemika) applied via a local (distance < 300 μm) perfusion pipette. Fluo-4 was excited at 475 nm, and its fluorescence emission was collected at 525/50 nm, and images were made using TILL Vision software (TILL Photonics).

The analysis was performed using custom-written macros in IGOR PRO (Wavemetrics). Images were opened in IGOR and, if necessary, motion stabilized using custom written registration routines, to remove movement artifacts in the extracted traces. Regions of interest (ROIs) were drawn over Fluo-4-labeled neurons and fluorescence intensities and percentage of responsive neuron were calculated. Neurons were only included in the Ca^2+^ analysis when they displayed a sharply increasing Ca^2+^ response to the high-K^+^ perfusion. The fluorescence intensities were normalized to the basal fluorescence at the onset of the recording for each ROI (ΔF/F_0_), and the % increase was analyzed. A peak was considered if the signal rose above baseline plus two times the intrinsic noise level.

### Immunofluorescence staining and cell counting

After live imaging, the tissue was put for 30 min at RT in fixative solution containing 4% paraformaldehyde in PBS and then washed three times for 10 min in PBS. For immunostaining, tissues were incubated for 2 h at RT in blocking buffer containing 0.1% Triton X-100, 2% goat serum, and 2% donkey serum in PBS and then incubated overnight at 4°C with primary antibodies. Nerve fibers and neurons were, respectively, labeled with chicken anti-neurofilament 200 kDa, NF200 (1:500; ab72996, Abcam) and mouse anti-panneuronal HuCD (1:500; A21271, Invitrogen). Tissues were then incubated for 2 h at RT with appropriate fluorescently labeled secondary antibodies [donkey anti-mouse Alexa Fluor 488 (A21202) and goat anti-chicken Alexa Fluor 594 (A11042), ThermoFisher]. After three washes in PBS, tissues were mounted on a slide and visualized under an epifluorescence microscope (BX 41 Olympus) to count neurons and ganglia. Neurons were counted based on the HuCD immunostaining, as we previously described (Cirillo et al., 2013). All counting was done in a blinded manner. Further, localization of HuCD protein in the nucleus and cytosol within a neuron was also evaluated to further characterize the morphology and the condition of the neuron (Desmet et al., 2014). Representative confocal images were then recorded using a Zeiss LSM 780 Meta confocal microscope (Cell Imaging Core, Katholieke Universiteit Leuven).

### Data and statistical analysis

Data are presented as median (range). Normality of data distribution was tested by D’Agostino and Pearson omnibus test. Outliers were identified by Grubb’s test. Data not normally distributed were compared by Kruskal–Wallis test, *p* < 0.05 was considered significant. Statistical analysis was performed with GraphPad Prism 8.04 (GraphPad).

## Results

### Tissue morphology and cellular integrity

**Figure 3.**
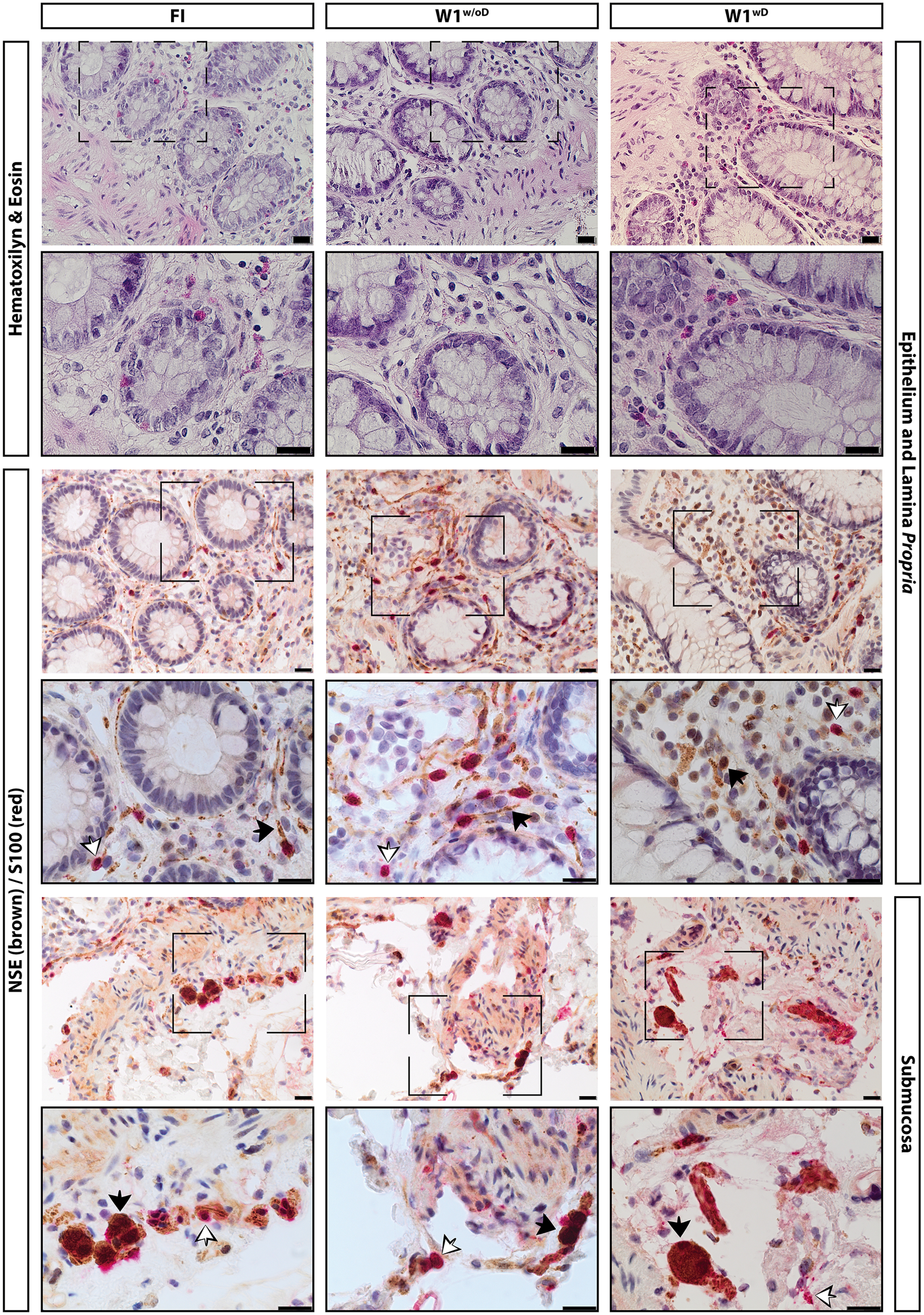
Representative images of H&E, NSE (black), and S100 (white) immunohistochemical stainings colonic biopsies, before cryopreservation conserved in NBM and after one-week cryopreservation with and without DMSO. H&E, NSE (brown), and S100 (red) images are shown as labeled on the left. The tissue sections (epithelium, lamina propria, or submucosa) are labeled on the right. The first column shows freshly isolated and fixed biopsies, second column shows biopsies fixed after one week of cryopreservation with NBM, third column shows biopsies fixed after one week of cryopreservation with NBM and DMSO. For each staining, the black dashed regions are shown magnified in the row below. Scale bars: 20 μm. White arrows point at S100-positive glial cells, and black arrows show NSE-positive neurons.

H&E staining showed intact mucosa, epithelium and lamina propria, crypts and enterocytes in each of the conditions tested: either freshly fixed or fixed and after one week of cryopreservation in NBM, with or without DMSO (Fig. 3). No morphologic differences were detected in cryopreserved-fixed colonic biopsies when compared with freshly fixed specimen, either with or without DMSO.

We confirmed our visual microscopic assessment by histologic scoring (McGowan et al., 2012) of H&E-stained sections. The histologic score did not reveal any significant differences between cryopreserved and fresh biopsy sections (Table 2).

**Table 2:** McGowan histologic scoring and eosin*-*positive cells counts

	*n*	No damage (grade 1–2)	Moderate damage (grade 3–4)	Severe damage (grade 5–6)
Fresh isolation	6	100%	0%	0%
W1*^w^*^/^*^o^*^D^	3	100%	0%	0%
W1*^w^*^D^	3	100%	0%	0%

Values are expressed as percentage (%)

Given the focus on specifically preserving neuronal viability, we first analyzed sections of colonic biopsies after cryopreservation, using immunohistochemistry to evaluate neuronal and glial morphology. Similar as with H&E staining, no changes in terms of cellular integrity of enteric neurons and/or glia were observed when comparing freshly fixed with cryopreserved-fixed tissue. In all conditions, NSE and S100-positive cells were detected in the lamina propria and in the submucosa, either scattered or contained in small ganglia (Fig. 3). Thus, we can assume that the cryopreservation process does not alter either the histology of the tissue or the presence of enteric neurons or glia.

### Protocol optimization

Aiming to set up the best conditions for tissue cryopreservation, different variants of the protocol were assessed (Table 3). Using live imaging, we found that biopsies are no longer viable after storing them simply for 6 h at 4°C in NBM (condition 1), hence, cryopreservation steps were introduced. Freezing biopsies directly at −20°C or −80°C never yielded any viable neurons after thawing (conditions 2 and 3). Therefore, the cryoprotectant agent DMSO was added to the medium in the following protocol optimization steps. Snap freezing the biopsies in liquid nitrogen with NBM and DMSO still did not yield viable neurons (condition 4), and neither did adding a previous cooling/freezing step of either −20°C or −80°C (conditions 5 and 6, respectively). In the final freezing conditions (7 and 8), which neurons were indeed able to survive, isopentane was used to achieve a gradual freezing at −80°C from 5 to 10 h, after which the neuronal tissues were transferred to liquid nitrogen.

**Table 3:** Cryopreservation conditions set up

Condition	Preservation medium	Cooling/freezing	W/WO isopentane	Storage	Responding cells (%)
1	NBM	4°C	-	4°C > 6 h	0%
2	NBM	−20°C	WO	−20°C	0%
3	NBM	−80°C	WO	−80°C	0%
4	NBM + 8% DMSO	Snap Liq N_2_	WO	Liq N_2_	0%
5	NBM + 8% DMSO	−20°C, 10 min	WO	Liq N_2_	0%
6	NBM + 8% DMSO	−80°C, 24 h	WO	Liq N_2_	0%
7	NBM	−80°C, 5–10 h	W	Liq N_2_	<50%
8	NBM + 8% DMSO	−80°C, 5–10 h	W	Liq N_2_	>50%

NBM, Neurobasal medium; DMSO, Dimethyl sulfoxide; Liq N2, Liquid nitrogen; W, with; WO, without.

Here, the condition with DMSO (8) yielded better results than without (7) DMSO (see Table 3 and results below). For the comparison of the different conditions in terms of how well neurons survived, we only used an artificial (high-K^+^) depolarization to test what percentage of neurons was responding. We aimed at having at least 50% of neurons responding to the high-K^+^ stimulus. It is important to highlight that stimulating with more specific agonists or drugs might result in a lower response rate.

**Figure 4.**
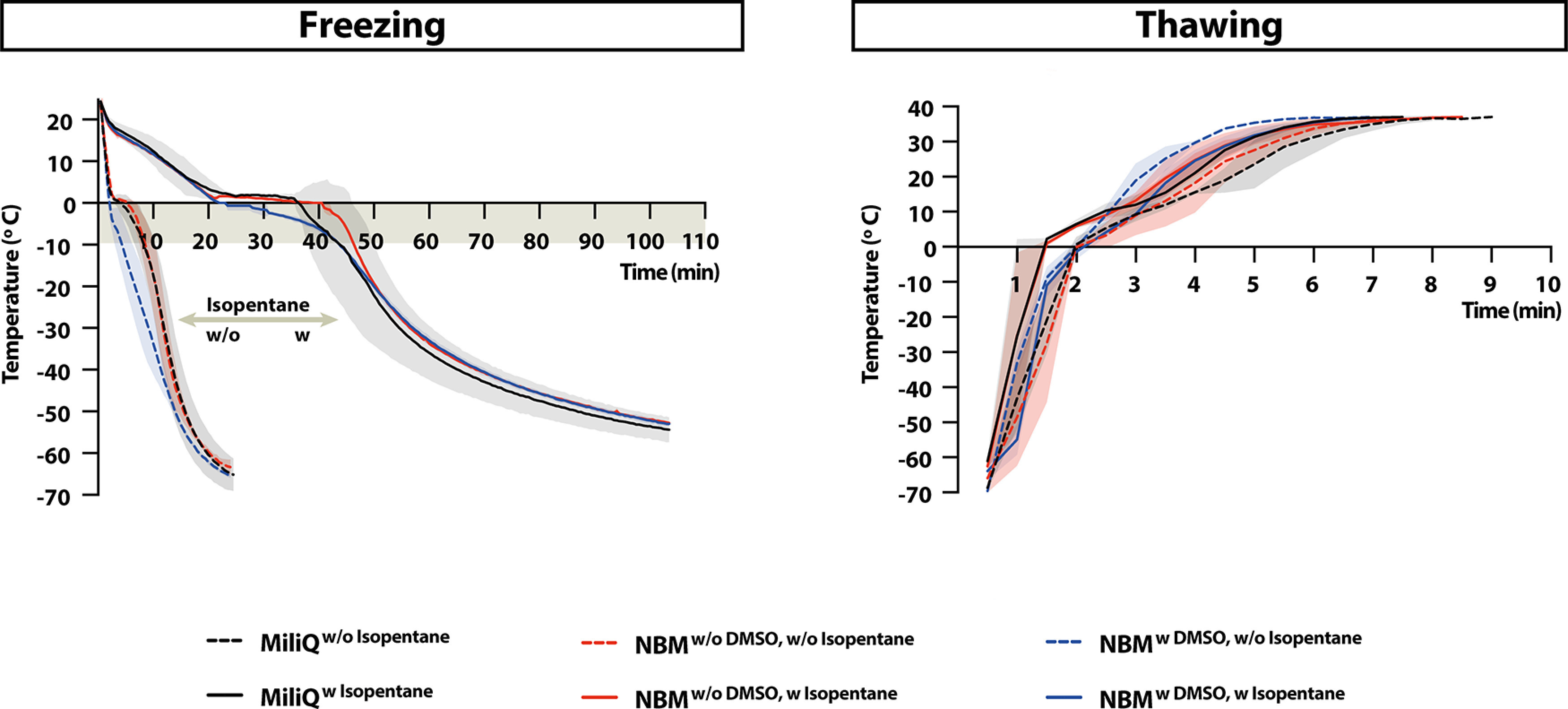
Temperature curves for freezing and thawing procedures. All conditions with isopentane full lines; without isopentane, dashed lines. MilliQ water in blue; NBM without DMSO in green; NBM with DMSO in orange. Note that isopentane slows the freezing process over 5-fold independent of the medium (gray double headed-arrow); furthermore, the addition of DMSO shifts freezing curves moderately to the left, specifically in the −5°C range (shaded bar). The effects of either DMSO or isopentane during thawing are less pronounced. DMSO, Dimethyl sulfoxide; NBM, Neurobasal medium; w, with; w/o, without.

Temperature recordings were performed for different conditions; including milliQ water with and without isopentane, and conditions 6, 7, and 8 as well as NBM without DMSO and isopentane. The use of isopentane flattened the freezing curve, avoiding snap freezing and favoring cell viability (Fig. 4; Table 3). Interestingly, the addition of DMSO specifically sped up freezing between 0°C and −10°C (Fig. 4, shaded bar).

### Quantification of enteric ganglia and neurons

**Figure 5.**
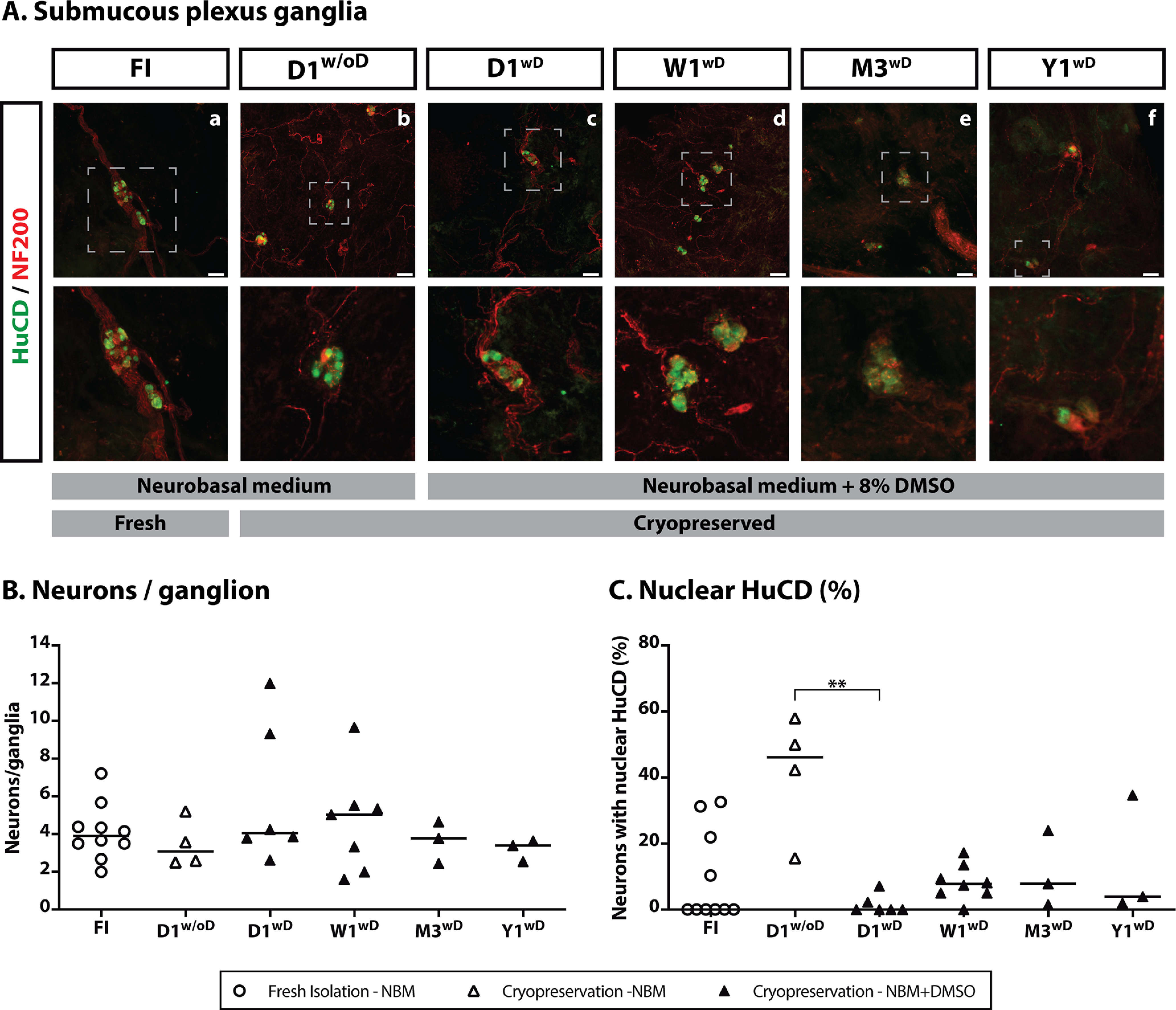
***A***, Representative images of immunohistochemical stainings of dissected submucous ganglia. Submucosal neurons (HuCD) in green and nerve fibers (NF200) in red. Immunostainings were performed either immediately (***a***) or after cryopreservation for different time periods: 1 d without (D1^w/oD^; ***b***) or with (D1^wD^; ***c***) DMSO, one week (W1^wD^; ***d***), three months (M3^wD^; ***e***) or one year (Y1^wD^; ***f***). Magnification of the selected ganglion (square) from top panel is in the bottom panel. Scale bar: 50 μm. ***B***, ***C***, Quantification of submucosal neurons. ***B***, Average number of neurons per ganglion. ***C***, Percentage of neurons with nuclear HuCD. The individual data points represent a subject for which all individual neuronal responses were averaged. Horizontal bars represent the median. Comparison between groups was performed using Kruskal–Wallis test, *p < 0.05, **p < 0.01.

The HuCD immunofluorescence staining did not reveal any difference in the number of ganglia per biopsy among groups [FI: 3.5 (7.5) ganglia/biopsy, D1^w/oD^: 4.3 (6) ganglia/biopsy, D1^wD^: 3.1 (8) ganglia/biopsy, W1^wD^: 8.9 (22) ganglia/biopsy, M3^wD^: 9.7 (6.8) ganglia/biopsy, and Y1^wD^: 6.7 (2) ganglia/biopsy; Fig. 5*A*]. Similarly, the number of neurons per ganglia was not different in dissected SMP postcryopreservation with DMSO [D1^wD^: 4.1 (9.4); W1^wD^: 5.0 (8.1); M3^wD^: 3.8 (2.2) and Y1^wD^: 3.4 (1.1) neurons/ganglia] when compared with fresh SMP [FI: 3.9 (5.2) neurons/ganglia]. Remarkably, these parameters were unaffected even if DMSO was left out [D1^w/oD^: 3.1 (2.7) neurons/ganglia; Fig. 5*B*], consistent with the histologic analysis and cellular integrity assessment, as described above.

A nuclear HuCD staining is indicative of poor neuronal health, as previously described (Desmet et al., 2014); therefore, we used the nuclear HuCD as a parameter to judge neuronal health. The percentage of neurons with nuclear HuCD labeling was increased in the tissue preserved without DMSO [D1^w/oD^: 46.2 (42.4)%], when compared with fresh SMP (FI: 0 (32.6) and to DMSO preserved tissue [D1^wD^: 0 (7.1)% (*p* = 0.009), W1^wD^: 7.8 (17.2)%, M3^wD^: 7.8 (22.5)%, and Y1^wD^: 3.9 (32.7)%; Fig. 5*C*]. The increase of nuclear HuCD may be indicative of cell degeneration after longer periods of cryopreservation.

### Response of enteric neurons to high-K^+^, selective nicotinic agonist DMPP and 5-HT

**Figure 6.**
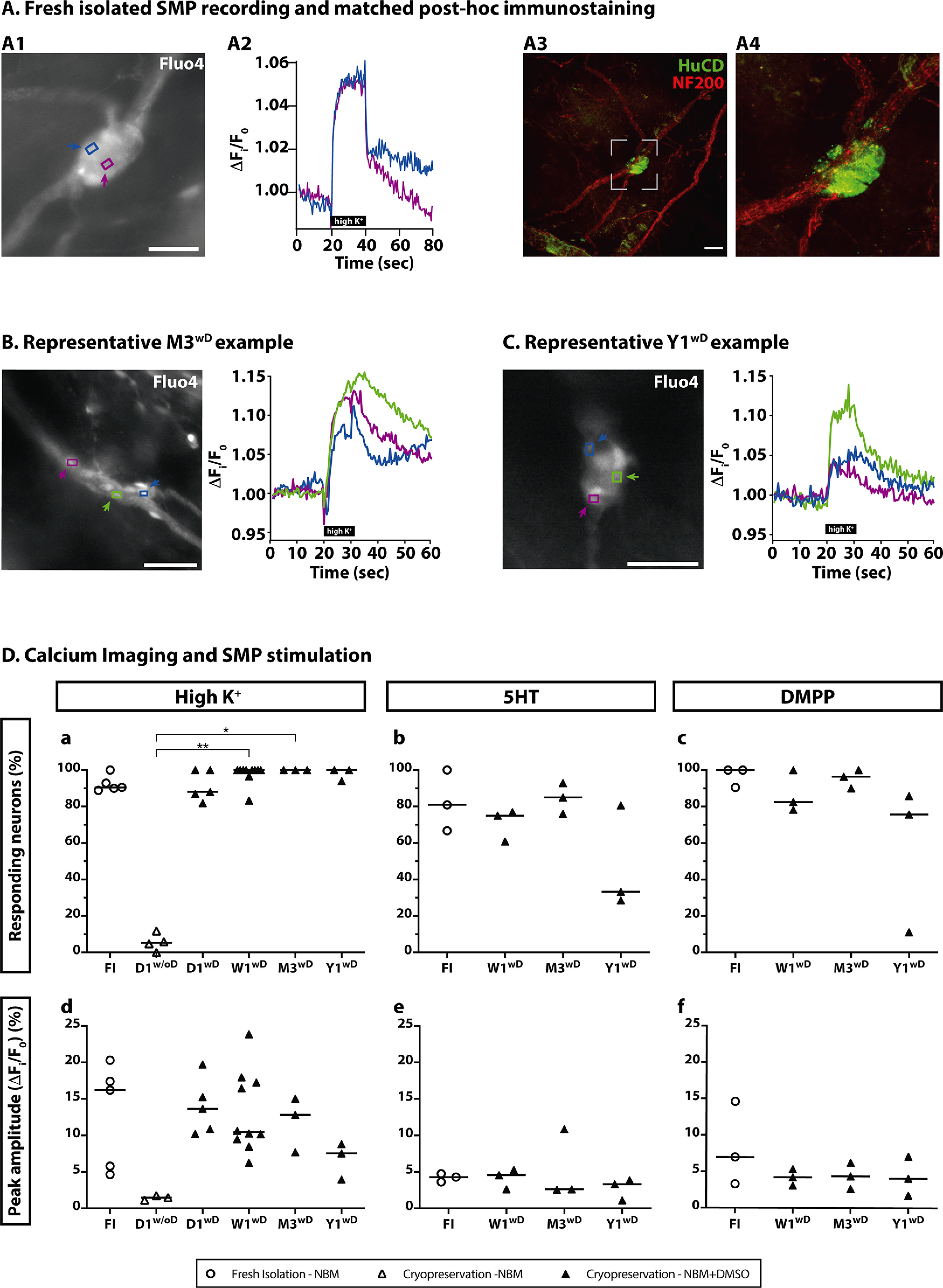
Ca^2+^ imaging in human biopsies. ***A***, FI SMP recording and matched *post hoc* immunostaining. ***A1***, Representative image of a SMP ganglion loaded with Fluo-4. Color-coded arrows point at two neurons, whose Ca^2+^ traces are shown in ***A2***. Scale bar: 50 μm. ***A2***, Normalized fluorescence profiles (ΔF/F_0_) of the two neurons marked in ***A***. ***A3***, The submucous ganglion (shown in ***A1***) fixed and immunostained for HuCD (green) and NF200 (red). ***A4***, Larger magnification of the dashed box. Scale bar: 50 μm. ***B***, Representative image of a SMP ganglion after three months of cryopreservation loaded with Fluo-4. ROIs mark three different neurons whose Ca^2+^ traces are shown on the right. The actual recording corresponds to Movie 1. Scale bar: 50 μm. ***C***, Representative image of a SMP ganglion after one year of cryopreservation loaded with Fluo-4. ROIs mark three different neurons whose Ca^2+^ traces are shown on the right. The actual recording corresponds to Movie 2. Scale bar: 50 μm. ***D***, Summary data of the neuronal responses to three different stimuli for six different conditions. Top row shows the percentage of responding neurons and bottom row the peak amplitude after stimulation with high-K^+^, 5-HT (serotonin) and DMPP (dimethylphenylpiperazinium). The individual data points represent a subject for which all individual neuronal responses were averaged. Horizontal bars represent the median. Comparison between groups was performed using Kruskal–Wallis test, *p < 0.05, **p < 0.01.

To determine neuronal viability in the cryopreserved and thawed tissues, we used Ca^2+^ imaging, as previously described (Cirillo et al., 2013). We applied high-K^+^ solution to depolarize neurons and analyzed their Ca^2+^ transients. Each ganglion contained 2–10 neurons (Fig. 6*A*-*C*; Movie 1, 2). Neurons within the ganglia are discriminated from glial cells by their size, round morphology together with a dimmer Fluo-4 loading. Moreover, glia cells do not respond immediately to high-K^+^, but only shortly after and as a consequence of neuronal depolarization (Boesmans et al., 2013).

Movie 1. Representative recording of high-K^+^ Krebs stimulation of the colonic ganglion Fluo-4 loaded after one year of cryopreservation shown in Figure 6. A 1-min recording at 2 Hz. Timescale shown at top left. Upon high-K^+^ Krebs stimulation (between second 20 and 30, as shown by the hK mark below the timescale), a ganglion response can be observed. Scale bars: 20 μm. 10.1523/ENEURO.0388-21.2021.video.1

Movie 2. Representative recording of high-K^+^ Krebs stimulation of the colonic ganglion Fluo-4 loaded after three months of cryopreservation shown in Figure 6. A 1-min recording at 2 Hz. Timescale shown at top left. Upon high-K^+^ Krebs stimulation (between second 20 and 30, as shown by the hK mark below the timescale), a ganglion response can be observed. Scale bars: 20 μm. 10.1523/ENEURO.0388-21.2021.video.2

The percentage of responding ganglia in tissues preserved without DMSO [D1^w/oD^: 5.3 (11.8)%] was substantially lower than in FI tissues [FI: 90.5 (11.1)% (NS)] or those cryopreserved in the presence of DMSO [D1^wD^: 88 (18.2)%, (NS), W1^wD^: 100 (16.7)% (*p* = 0.004) and M3^wD^: 100 (0)% (*p* = 0.02) and Y1^wD^: 100 (6.1)% (NS)]. No differences were found between FI SMP neurons response and those preserved with DMSO, independently of the cryopreservation period (*p* > 0.05; Fig. 6*Da*).

A similar observation was made when analyzing the Ca^2+^ transient amplitude of the responding neurons. A lower, but statistically not significant (*p* = 0.08), peak is observed in tissues preserved without DMSO [D1^w/oD^: 1.5 (0.6)%] when compared with FI tissues [FI: 16.2 (15.6)%] as well as when compared with DMSO-cryopreserved tissues [D1^wD^: 13.7 (9.5)%, W1^wD^: 10.4 (17.6)%, M3^wD^: 12.8 (7.3)%, and Y1^wD^: 7.5 (4.9)%]. Again, no differences were found between neuron response amplitudes in FI SMP and those preserved with DMSO, independently of the cryopreservation period (*p* > 0.05). However, a slight decrease in the peak amplitude is observed with longer cryopreservation duration (Fig. 6*Dd*). These results suggest very limited survival after cryopreservation without DMSO.

In line with previous studies (Cirillo et al., 2013, 2015) and to further characterize neuronal responses to specific stimuli (nicotinic and serotonergic agonists), DMPP and 5-HT were perfused onto the SMP. Neuronal responses in FI and DMSO-cryopreserved tissues were analyzed at different time points (W1^wD^, M3^wD^, and Y1^wD^).

In FI SMP, we found that 81 (33)% and 100 (9.5)% of the neurons responded to 5-HT and DMPP, respectively, with an average Ca^2+^ transient amplitude of 4.3 (1.1)% for 5-HT stimulation and 7 (11.3)% for DMPP. No significant differences were observed between FI and cryopreserved SMP at different time points neither when stimulating with 5-HT [W1^wD^: 75 (16.1)%, M3^wD^: 85 (16.9)%] nor with DMPP [W1^wD^: 82.5 (21.7)%, M3^wD^: 96.4 (10)%; *p* > 0.05]. Nonetheless, a decrease in responsiveness is observed after one year of cryopreservation in both 5-HT [Y1^wD^: 33.3 (52.1)%] and DMPP [Y1^wD^: 75.6 (74.6)%; Fig. 6*Db*,*c*]. The same pattern is observed when analyzing Ca^2+^ transient amplitudes: no significant differences were found comparing FI [5-HT: 4.3 (1.2)%, DMPP: 3.3 (11.3)%] with cryopreserved SMP after 5-HT stimulation [W1^wD^: 4.6 (2.6)%, M3^wD^: 2.6 (8.3)%, and Y1^wD^: 3.3 (2.8)%] nor after DMPP stimulation [W1^wD^: 4.2 (2.3)%, M3^wD^: 4.4 (3.6)%, Y1^wD^: 4.1 (5.4)%; Fig. 6*De*,*f*]. The response of nerve cells to DMPP and 5-HT show the conservation of functional neurotransmitter receptors after the cryopreservation process.

## Discussion

This study was undertaken to tackle a major challenge in biomedicine: the availability of human primary neurons suitable for research. We describe a protocol that substantial advances the possibilities to use human nerve tissue in assays that require live neurons. Our results clearly show the possibility of performing live nerve recordings in primary neurons of the ENS of living subjects after cryopreservation even up to one year.

In order to translate (neuro)scientific results obtained from animals into realistic and reliable patient data, the use of human viable samples is mandatory (Schemann, 2011). Laboratory animals have been and are routinely used to study not only physiology and pharmacology but also pathologic aspects of neurologic disorders. Although these approaches have provided invaluable information that helps understanding molecular mechanisms, biochemical pathways, pathogenesis and etiology of many human diseases, they have crucial limitations: (1) the interpretation and translation of the results are often difficult because of species-related differences; (2) animal models do not always exactly recapitulate key aspects of human diseases, which again, hampers the correct interpretation of the results. Human nerve tissue is very scarce. For example, brain tissues can be used only postmortem for observational studies since biopsies taken from patients is far from a custom method, especially because of the high risks to the patients (Constantini et al., 2013).

One recent and promising example to obtain live human neurons is the use of fibroblasts isolated from skin biopsies (Takahashi and Yamanaka, 2006). Skin fibroblasts can be reprogrammed to generate induced-pluripotent stem cells by using a cocktail of pluripotency factors (Takahashi and Yamanaka, 2006; Ambasudhan et al., 2011; Caiazzo et al., 2011; Pang et al., 2011). By applying different protocols, induced-pluripotent stem cells can successfully generate human neurons, which allow setting up *in vitro* experiments. Despite the great innovation introduced by this approach, crucial aspects need to be considered: (1) fibroblasts need to be artificially manipulated through at least (or alternatively) two steps before becoming neurons; (2) the success rate of neuronal generation is quite low (Qiang et al., 2013); and (3) neurons often remain in an immature state even after long differentiation periods. Evidence has demonstrated that the human ENS may serve as a worthy source of true primary neurons (Lebouvier et al., 2008; Pouclet et al., 2012; Cirillo et al., 2013, 2015; Wouters et al., 2016; Desmet et al., 2017), void of the possibility that molecular reprogramming has altered their fate.

This specific study explored whether it is possible to maintain human primary neurons alive after cryopreservation of the nerve tissue taken from the ENS.

First and foremost, the conditions for an optimal nerve tissue cryopreservation were established. Given the high enzymatic and immunologic activity, intestinal biopsies face rapid degradation on their removal. Therefore, a quick separation of the nerve layer (namely, the SMP) from the epithelium is of utmost importance for the neuronal survival, avoiding the production of proinflammatory and proapoptotic factors. No successful preservation was achieved with any of the freezing conditions at temperatures above −25°C since enzymatic activity is, although slowed down (Bakhach, 2009), still present. The same outcome was observed when biopsies were directly stored at −80°C, since at that temperature preserved cells are not stable, presumably because of traces of unfrozen media (Mazur, 1984). It was thus decided to use a cryoprotectant agent. First, the addition of DMSO was explored, but this was not sufficient for assuring tissue viability in neither snap freezing nor with freezing periods at −20°C or −80°C. DMSO is an intracellular active cryoprotectant able to reduce the formation of cellular crystals. Nevertheless, when cooling is rapid, the water inside the cells tends to generate small thermodynamically unstable crystals that aggregate during the warming process leading to cell death (Mazur, 1984).

Hence, the optimal cooling speed is a crucial aspect when it comes to ensuring and maximizing cell survival. This window is known as “transition zone” and reflects a balance between avoiding the formation of intracellular ice because of rapid cooling of the medium and the cellular dehydration because of slow cooling (Mazur, 1984; Bakhach, 2009). To target the optimal transition zone, we included isopentane in the freezing protocol, offering a controlled cooling to −80°C through a rate of cooling of approximately −1°C per minute. In none of the conditions where freezing was done without isopentane, we observed any viable neurons. As expected, we found that the combined use of a cryoprotectant agent such as DMSO (with specific effects at the −5°C mark), and the controlled slow freezing speed using isopentane (Fig. 4, arrow, rightward shift), is crucial to preserve the viability of the nerve tissue.

Significant differences were observed when comparing 1 day frozen biopsies with or without DMSO. While cell responsiveness was around 90% for D1^wD^, at D1^w/oD^, only 5% responded to high-K^+^, with a lower amplitude peak of the few neurons responding. Because any cryopreservation condition without DMSO performed poorly in viability checks with high-K^+^, nicotinic and serotonergic signaling was only tested in DMSO containing conditions. Furthermore, we did not analyze the 1D condition as we assumed that in this is not a realistic future situation, there is very little benefit of keeping a biopsy deeply frozen for only 1 day. Moreover, in line with the results of high-K^+^ depolarization in all conditions analyzed, we can assume that they as well will be preserved after 1 day of freezing.

The percentage of neurons with nuclear HuCD was significantly higher in biopsies preserved without DMSO, indicative of poor neuronal health, as previously described (Desmet et al., 2014). Whole mucosal architecture was not affected by cryopreservation either with or without DMSO, as evidenced by the H&E analysis, suggesting that the formation of intracellular crystals mainly affects single-cell viability and function, while other parameters classically detected in fixed tissue remain unharmed.

Neuronal viability after cryopreservation was determined at different time points and defined as the ability of neurons to be depolarized by high-K^+^ (Cirillo et al., 2013). The percentage of responding neurons in all DMSO cryopreserved-conditions analyzed was above 90%, similar to the cellular viability after fresh isolation, and consistent with our previous observations (Cirillo et al., 2013, 2015; Desmet et al., 2017). The percentage of neurons displaying nuclear HuCD was equally low in all groups. Interestingly, we observed a slight decrease, although without significant difference, in transient amplitude in neurons after one-year cryopreservation. These results suggest that the one-year time point might be looked at as maximum extent for cryopreservation of human nerve tissue, at least with our protocol. It could be interesting to follow-up nerve tissue availability beyond one year; however, for the applications that we envision at present, the one-year time point is already as an important achievement.

This study also reveals that not only the physiological functioning of neurons within the nerve tissue is preserved but also their specific responses to major nicotinic and serotonergic agonists. Again, a slight reduction of the percentage of responding neurons is observed after one year of cryopreservation. Nonetheless, the percentage or peak amplitude did not show differences when compared with the freshly isolated nerve tissue. We are aware of the small sample size investigated for the longer cryopreservation conditions (three months and one year). However, the data of peak amplitude and the percentage of responding neurons recorded after high-K^+^ depolarization indicate a reliable consistency. Importantly, this study may establish an inflection point for human primary neurons imaging, becoming a launch pad for translational research.

Before this study, live imaging studies in human primary neurons would require a complex sequence of steps and events, which may only be accessible to those research institutes associated to a hospital complex, together with onsite expertise in dissection skills and imaging techniques.

From a practical point of view, our protocol can also be advantageous for its ethical aspects. It is known that the ethical consent for biopsies collection from endoscopic procedures allows to take several biopsies per patient, including some for use by pure research groups. The possibility of successfully cryopreserving human nerve tissue, would alleviate the workload, improving organization and maximizing optimal use of biopsies and resources.

In conclusion, this study substantially advances the possibilities to use primary human neurons in neuroscience research and renders human ENS samples valuable and easily-available experimental tools for daily work. Our protocol also favors the expansion of research networks, broadening collaborations among institutes with different expertise, by providing a unique tool to those research institutes not associated with hospital centers. We can imagine that cryopreserved samples may, from now on, be shipped worldwide, favoring more intense investigations.

## Synthesis

Reviewing Editor: Eran Perlson, Tel Aviv University Decisions are customarily a result of the Reviewing Editor and the peer reviewers coming together and discussing their recommendations until a consensus is reached. When revisions are invited, a fact-based synthesis statement explaining their decision and outlining what is needed to prepare a revision will be listed below. The following reviewer(s) agreed to reveal their identity: James Sleigh.

Experimentation with human tissues and cells is required to confirm findings from animal models. Primary human neurons are particularly difficult to obtain due to the short time frames that need to be cultured to remain viable. In this study, the authors present a method for generating human neuronal cultures from the intestine after cryopreservation. They identify conditions to aid cell health and function preservation during freezing and confirm cellular viability and function post-thawing after a week and up to a year in storage. Notably, the displayed protocol does not allow only to use of the cryo-preserved tissue for staining but also for live imaging upon thawing, as indicated by neuronal activity using calcium imaging. The combination of obtaining human patient samples and long-term preservation of live neurons allows an elegant solution for studying human neurons that many labs can utilize in the future. Overall, the study is clear, logical, and well presented.

Still, before accepting this manuscript for publication, some clarifications for the comments below are needed:

1. How were the neurons per ganglia determined? No clear explanation was provided.

2. At line 255, the authors describe the choice of the cryo-preservation protocol as a solution for lack of neuronal viability after storage of 6h at 4oC. They analyzed this using live imaging methods but did not display the data indicating those samples were not viable after this relatively short time. Also, for the other conditions in Table 3, or at least for the NBM vs. NBM+8%DMSO conditions, it will be better to show some quantification or movies indicating neuronal viability other than no/low/good depicted in the current text. How can one decide if the calcium activity of neurons is low or good? Is it the percentage of activated neurons? Or action potential frequency? This part should be further explained.

If applicable, another possible approach to strengthen their findings is to quantify cell viability using a simple fluorescent reporter, such as Hoechst stain or TUNEL assay.

3. The NF200 staining is barely visible in Figure 5 and should become clearer. For instance, the Y1WD panels on the right look to lack NF200 completely. In fact, in Figure 5A - The authors use neuronal marker NF200 to visualize neurite morphology and health. Indeed, the data indicate specific staining. However, the images show that only in the F1, D1w/oD, and D1wD are healthy NF200 stained neurites, while the other images from older periods display fragmented to non-existing NF200 signals. This might show neuronal degeneration even after one week of preservation. The authors should display more representative images or explain the lack of NF200 signals in their older samples.

4. It would be helpful to the reader if the meaning of nuclear HuCD staining were explained when first introduced in the Results section.

Figure 5B - The authors assess the neuronal marker HuCD nuclear localization to show that DMSO addition improves neuronal viability. However, the displayed images are without a nuclear marker, such as DAPI, so it is hard to understand how this percentage of nuclear-localized HuCD was calculated. Also, the HD staining is somewhat saturated, causing difficulty in judging the differences between various conditions. Specifically, the D1w/oD staining looks similar to other conditions despite it should have increased nuclear colocalization according to the quantification.

Please also make HuCD uniform - sometimes HuC/D is used instead.

4. In figure 6A, it is hard to estimate the quality of the calcium imaging movies. It can be helpful to attach an additional movie figure or display several time-frame images in higher magnification to visualize how single-cell fluorescence was determined.

5. In figure 6C, it is described in the text that D1w/oD condition has a lower peak amplitude. However, there is no indication of significance in the attached graph.

6. A general comment - It might be better to add a sentence or two at the end of each results section to explain the author's interpretation of the data.

7. This reviewer does not identify the phrase “life-imaging” used by the authors at several instances throughout the manuscript (also in the significance statement). I would suggest changing it to the familiar, known term “live-imaging.”

Typo Figure 1: “Life Imaging"

8. In Figure 4, there is no indication for the X-axis (presumably time in minutes) on the figure image. Also, “NBM who DMSO w Isopentane” should be “NBM w/o DMSO w Isopentane.”

9. Line 430 - the word “also” repeats twice.

10. Supplementary Figure 1 legend - the arrows in the figure are black and white, while the writing indicates red and brown.

11. In the FI SMP DMPP data - it is unclear how the three values can have a median of 100% (line 317, Figure 6C, panel c) when one of the values is less than 100%.

12. Line 274: saying that DMSO “sped up freezing around -5˚C” is imprecise - a time value would be more appropriate.

13. Please provide details of the posthoc tests performed after Kruskal-Wallis tests to ascertain the P values.

14. Please provide catalog numbers and working concentrations for all antibodies.

15. Please check the arrow colors in the legend of Figure 4 and Supplementary Figure 1.

Typos

• Line 70: “has been has been"

• Line 80: “to make to"

## Author Response

Response to editorial board and reviewers

Thank you very much for the opportunity to resubmit our manuscript in a revised version based on the editors’ and reviewers’ comments. We would like to thank reviewers and editorial office for their constructive comments and suggestions to improve our manuscript and for understanding the complexity and limitations of this human study. We believe that the paper has improved as a result.

We have responded point-by-point to the comments below (in italics) and have marked the changes in the revised manuscript

1. How were the neurons per ganglia determined? No clear explanation was provided.

Neurons per ganglia are determined based on a post-hoc immunostaining using a HuCD antibody. This panneuronal marker is known to label neuronal cell bodies and/or nuclei, but not neuronal processes, which allows accurate counting of neurons. The method, as used also in our previous studies (Cirillo 2013) is widely used in the enteric and central nervous system as reliable neuronal identifier.

In the revised text, we provided this explanation. See line 220: To quantify neurons HuCD cells were counted Neurons were counted by using HuCD immunostaining as we previously described (Cirillo 2013) and line 278: Immunofluorescence analysis HuCD immunofluorescence staining did not reveal any difference within in the number of ganglia per biopsy among groups.

2. At line 255, the authors describe the choice of the cryo-preservation protocol as a solution for lack of neuronal viability after storage of 6h at 4oC. They analyzed this using live imaging methods but did not display the data indicating those samples were not viable after this relatively short time. Also, for the other conditions in Table 3, or at least for the NBM vs. NBM+8%DMSO conditions, it will be better to show some quantification or movies indicating neuronal viability other than no/low/good depicted in the current text.

As requested by the reviewer, we provide additional details related to the assessment of viability or non-viability of neurons. The ‘no/low/good’ qualifier was based on the percentage of neurons activated by high-K+, with 0% responders for conditions 1 to 6 (no viability), 5.3% responding neurons was for condition 7 (low viability), and above 80% for condition 8 (good viability). However, we agree that this qualitative parameter is not a good description of how neuronal viability was assessed. To avoid confusion, this qualitative parameter was changed, and “viability” was replaced by the percentage of responding cells, which is much more quantitative.

How can one decide if the calcium activity of neurons is low or good? Is it the percentage of activated neurons? Or action potential frequency? This part should be further explained.

In our analysis, using custom-written algorithms in Igor (Wavemetrics), we consider intracellular calcium concentration peaks to be responses if they exceed the baseline plus 5 times the intrinsic noise level. As soon as one such peak is detected, the neuron is considered a responder, and counts towards the percentage of responsive neurons per ganglion.

If applicable, another possible approach to strengthen their findings is to quantify cell viability using a simple fluorescent reporter, such as Hoechst stain or TUNEL assay.

We understand the remark related to the staining with Hoechst or with the Tunel assay, however we prefer to use a staining (Fluo-4 AM) that highlights viability and can be immediately used to assay neuronal activity, rather than a staining for damage. Hoechst would indeed show labeling of nuclei in cells that have leaky membranes and Tunel is definitely a useful technique to identify DNA fragmentation derived from apoptosis. Although it may help us to identify the most severely damaged cells and apoptotic cells, we expect many of the neurons that did not survive (in conditions 1-6) are just damaged because of the physical process of freezing and thawing, and have not necessarily triggered apoptosis.

3. The NF200 staining is barely visible in Figure 5 and should become clearer. For instance, the Y1WD panels on the right look to lack NF200 completely. In fact, in Figure 5A - The authors use neuronal marker NF200 to visualize neurite morphology and health. Indeed, the data indicate specific staining. However, the images show that only in the F1, D1w/oD, and D1wD are healthy NF200 stained neurites, while the other images from older periods display fragmented to non-existing NF200 signals. This might show neuronal degeneration even after one week of preservation. The authors should display more representative images or explain the lack of NF200 signals in their older samples.

As requested by the reviewer and to strengthen the message, in the revised manuscript we have provided better images of the NF200 staining. Please, see the updated figure 5 in the manuscript. It has to be noted that not all ganglia have the same NF200 labeling with thick fibers surrounding the ganglia. Therefore the variation in NF200 staining in the displayed images is not a sign of deterioration due to the preservation procedure. Therefore we added below some extra examples of NF200 staining of other areas in tissues after different times of cryopreservation.

4. It would be helpful to the reader if the meaning of nuclear HuCD staining were explained when first introduced in the Results section. Figure 5B - The authors assess the neuronal marker HuCD nuclear localization to show that DMSO addition improves neuronal viability. However, the displayed images are without a nuclear marker, such as DAPI, so it is hard to understand how this percentage of nuclear-localized HuCD was calculated. Also, the HD staining is somewhat saturated, causing difficulty in judging the differences between various conditions. Specifically, the D1w/oD staining looks similar to other conditions despite it should have increased nuclear colocalization according to the quantification. Please also make HuCD uniform - sometimes HuC/D is used instead.

We agree with the reviewer that this HuCD localization needs more explanation and apologize for the saturated HuCD images. In the corrected version of the manuscript, we have provided better quality images that allow to appreciate the localization of this protein. The nuclear localization of HuCD can be seen as in indicator for decreasing neuronal health (Desmet, NGM 2014). Here below we show, some representative images of HuCD staining, combined with DAPI for clarity, in which the difference between cytosolic (c) vs. nuclear (n) localization of HuCD can be clearly seen. We have made the notation for ‘HuCD’ uniform throughout the text.

The text in the manuscript (line 289) has changed as follows: Nuclear HuCD staining is indicative of poor neuronal health, as previously described (Desmet et al., 2014), therefore we used the nuclear HuCD as a parameter to judge neuronal health. The percentage of neurons with nuclear HuCD labeling was increased in the tissue preserved without DMSO (D1w/oD: 46.2 (42.4) %), when compared to fresh submucosal plexus (FI: 0 (32.6) and to DMSO preserved tissue (D1wD:0 (7.1) % (p-value=0.009), W1wD: 7.8 (17.2) %, M3wD: 7.8 (22.5) % and Y1wD: 3.9 (32.7) %) (Figure 5C).

4. In figure 6A, it is hard to estimate the quality of the calcium imaging movies. It can be helpful to attach an additional movie figure or display several time-frame images in higher magnification to visualize how single-cell fluorescence was determined.

To help judging the quality of the calcium imaging, we have uploaded as supplementary material two representative movies, one of a ganglion that was stored for 3 months and another one of a tissue that was kept frozen for a year. One time-frame of each of these movies, together with the depolarization traces was added as panel B and C in the revised figure 6. We hope that the fluorescence changes that can be seen in the movies are helpful to appreciate the effects of high-K+ depolarization.

5. In figure 6C, it is described in the text that D1w/oD condition has a lower peak amplitude. However, there is no indication of significance in the attached graph.

The Kruskal-Wallis test p-value is 0.08, but due to the small sample size we cannot make any statistically valid claims. Therefore we decided to just describe in words the fact that without DMSO preservation is much worse. We have added the statistical information in the text (line 310).

6. A general comment - It might be better to add a sentence or two at the end of each results section to explain the author’s interpretation of the data.

A short explanation of the data interpretation has been added at the end of each results section.

7. This reviewer does not identify the phrase “life-imaging” used by the authors at several instances throughout the manuscript (also in the significance statement). I would suggest changing it to the familiar, known term “live-imaging.” Typo Figure 1: “Life Imaging”

We apologize for the apparent claim as if we would be imaging ‘life’, ‘live’ is indeed what we meant. All typos have been corrected.

8. In Figure 4, there is no indication for the X-axis (presumably time in minutes) on the figure image. Also, “NBM who DMSO w Isopentane” should be “NBM w/o DMSO w Isopentane.”

The indication for the X axis in figure 4 has been added (time (minutes)) and the w/o typo has been corrected.

9. Line 430 - the word “also” repeats twice.

We have corrected this mistake.

10. Supplementary Figure 1 legend - the arrows in the figure are black and white, while the writing indicates red and brown.

Arrow colors have been corrected in the text (red and brown).

11. In the FI SMP DMPP data - it is unclear how the three values can have a median of 100% (line 317, Figure 6C, panel c) when one of the values is less than 100%.

It is because values are expressed as median and not as mean because of the lack of normality due to the small sample size. While the mean expresses the arithmetic average, the median is the value separating the higher half from the lower half of the values. With the values being, in this case, 90.5, 100 and 100, median is 100.

12. Line 274: saying that DMSO “sped up freezing around -5˚C” is imprecise - a time value would be more appropriate.

We have corrected this point.

The text now reads in line 276: Interestingly, the addition of DMSO specifically sped up freezing around -5ºC between 0 and -10ºC (see shaded bar in Figure 4).

13. Please provide details of the posthoc tests performed after Kruskal-Wallis tests to ascertain the P values.

Dunn’s multiple comparison tests performed after Kruskall-Wallis are shown in the following table:

14. Please provide catalog numbers and working concentrations for all antibodies.

The lacking information has been added to the text.

15. Please check the arrow colors in the legend of Figure 4 and Supplementary Figure 1.

Color arrows in Supplementary Figure 1 have been corrected and also the “isopentane” arrow in the left panel has been changed.

Typos

• Line 70: “has been has been”

• Line 80: “to make to"

These typos have been corrected.
